# The Good of the Cause

**Published:** 1886-09

**Authors:** 


### THE GOOD OF THE CAUSE.

The Constitution of the Texas State Medical Association recites that the objects of said Association are "to organize the medical profession of the State in the most efficient manner possible,—to encourage a high standard of professional qualifications and ethics; and *to promote professional brotherhood.*" [Italic ours, Ed.]

The avowed mission of this Journal is, and ever has been, the organization of the regular profession of the State,—the building up of the State Medical Association, and the advancement of the best interests of legitimate medicine in Texas. We have striven to this end, and we rejoice in the prosperity of the Association. We love it; and when we have seen,—on more than one occasion,—the occurrence of events which appeared to us detrimental to its best interests,—at the risk of having our motives questioned, we have pointed it out, and advised its correction. Such was the Journal's criticism of the Dallas meeting.

Actuated by such regard for the future and permanent welfare of the Association, we expressed the opinion that theological discussions in the annual address of the President are out of place, and deprecated by the profession. Feeling well assured that a repetition of it would create dissensions and jealousies,—and, in the end, lead to the disruption of the Association,—as faithful watchmen on the tower, we sounded the alarm. Contrary to our expectations, we have fared much like the stranger did, who undertook to adjust a family difficulty.

There is nothing more absurd than the attempt to explain plain English, (unless it is to have to explain a joke), and nothing more unpleasant than personal explanations.

While our course in the premises has, by many prominent members, been appreciated and commended,—our language pronounced "courteous and conciliatory, yet of no uncertain sound ;"—while some have recognized in the much talked of editorial a sincere desire to preserve harmony, and, therefore, a service to the Association,—others have misconstrued our language,—impugned our motives, and accused us,—even, of *infidelity* ! assigning to us, meantime, all manner of unworthy motives, as instigating us to a "personal attack" on two of the Ex-Presidents—our personal friends !

While we are not indifferent to these acts of injustice, we can not do otherwise than bear them as best we may. The insinuations that the article in question was the outgrowth of any personal feeling on our part, of pique or resentment, are too absurd even to notice; but we feel called upon to correct the misapprehension as to our own religious sentiments. We were careful to express none, in the article referred to, yet, with a strange inconsistency, some of our friends have assumed that we are opposed to the Christian religion! We will say, once for all, the assumption is unwarranted, and, certainly, not justified by any line or sentence in our article of July. We also take occasion to disclaim any intention, or desire, to reflect on Drs. Brown and Becton. For those distinguished gentlemen we have, personally. the highest regard, and a respect for their religious principles. We have attributed to these gentlemen simply an error of judgment in giving utterance to religious sentiments in the association meetings, however much they may have felt justified by antecedent remarks of certain members in the course of certain essays, on former occasions. We differ with them as to the justification. Two wrongs do not make a right. While we approve, most cordially, of the practice of invoking the Divine Blessing on the labors of the Association, in the discussion of moot theological points, we recognize a dangerous and fruitless innovation; something entirely beyond the object and purposes for which the Association was created; and *it matters not who started it, it should cease,* at once, and forever! Especially should this be insisted upon, when the President, assuming to speak for, and in the name of the Association, pledges the members to the Christian, or any other faith, and denounces as "misguided" those who dissent from his position with reference to man's origin. We would advocate a broader spirit of toleration, were the discussion of the subject legitimately a part of the Association's programme; for, we hold that such a course and such language are not calculated to promote and foster "professional brotherhood" to any great extent, but rather to alienate the members, one from another, and all from the meetings.

In regard to the "views and opinions" expressed by Dr. Becton, in his address, and which THE JOURNAL said were not indorsed by the members, we had no reference whatever to his religious opinions, and so stated. The declaration which we challenged was, that "the great body of the profession" [meaning, of the Association, except those few "misguided" ones], KNOW that the doctrines of such men as Darwin and Huxley are withering, blighting as the winters frost,—that they clip the wings of noble aspiration, and damn the soul hereafter." We asserted that such opinions were not held by the association as a body—that the members have no such knowledge.

With this we dismiss the subject. We have the good of the cause too much at heart to sit idly by and witness the introduction of what we conceive to be an element of discord into the affairs of the Association,—a subject subversive of its best interests, and tending to its ultimate destruction, without raising our voice in warning. We have spoken without fear or favor, and having an approving conscience, we feel that we have discharged a duty, which, in time, will bring its own reward.

